# Interior Design: A New Perspective in Supportive Care of Patients with Acute Onset of Debilitating Diseases

**DOI:** 10.1089/pmr.2021.0031

**Published:** 2021-12-27

**Authors:** Davide Mauri, Eleftherios Kampletsas, George Smyris, Lampriani Tsali, Periklis Tsekeris, Haralampos Harissis, Konstantinos Kamposioras, Maria Tolia, Thomas Hyphantis, Panagiotis Ntellas, Ioanna Gazouli, Georgios Zarkavelis, Leonidas Mavroeidis, Anna-Lea Amylidi, Nanteznta Torounidou, Aristeidis Gogadis, Joanna Nixon

**Affiliations:** ^1^ Department of Medical Oncology, Psychiatry, Radiotherapy, and Surgery, University Hospital of Ioannina, Ioannina, Greece.; ^2^ Department of Architectural Engineering, Architecture Faculty, University of Ioannina, Ioannina, Greece.; ^3^ PACMeR, Department of Evidence Based Medicine, Athens, Greece.; ^4^ Department of Medical Oncology, The Christie NHS Foundation Trust, Manchester, United Kingdom.; ^5^ Department of Radiotherapy, School of Medicine, University of Crete, Heraklion, Greece.; ^6^ Department of Radiotherapy, Scottish Sarcoma Network (SSN) UK Chair NCRIHN Epidemiology and Survivorship Subgroup, The Beatson West of Scotland Cancer Centre, Glasgow, United Kingdom.

**Keywords:** aging population, debilitating diseases, functional indoor space, interior design, medical care, supportive care

## Abstract

**Background:**

Upon the onset of a debilitating rapidly evolving condition (such as cancer or a rapidly progressing myopathy, neuropathy, respiratory disease, or a severe traumatic injury), individuals have limited time to find a new home or make radical structural modifications in their residence. How the affected patients can continue sharing the same house with their families, while meeting their own special requirements, is thus rising as a critical issue. Household and daily routine rearrangements, either temporary or permanent, may be necessary, to ameliorate the life of patients with impairments, lasting for months or even years.

**Objectives:**

Interior design may provide a highly efficient “living” palliation for debilitating medical conditions directly at patients' home-site.

**Methods:**

Research of relevant literature, using keywords “debilitating conditions,” “home care,” “end of life care,” “care of advanced cancer patients,” “care of patients with mental disorders,” “home care of covid-19 affected patients,” and “care of patients with degenerative illnesses.”

**Results:**

We found that patients and their relatives may not be aware of the probable interior design solutions to their daily life challenges, imposed by a disease-related impairment. In parallel, interior design experts may equally be unaware of these issues, as well as of who needs the available solutions.

Similarly, medical and architectural sciences are not connected, eventually failing to meet patients' everyday needs.

**Conclusions:**

Interior architecture and health scientists are called to cooperate, aiming to provide a highly efficient and meaningful support to patients and families affected by unforeseen debilitating medical conditions.

## Introduction

Interior design is the art and science of making indoor space functional, healthier, aesthetically attractive, and safe by determining space requirements and selecting both decorative and essential living items. The science of interior design has an established and long-lasting tradition in medical care, addressing the need for vital health infrastructures such as hospitals, clinics, and ambulatory departments.^1,2^ During the past decade, interior design applications are expanding to the home-care setting due to the globally aging population. Between 2015 and 2050, the proportion of the world's population >60 years old will nearly double, increasing from 12% to 22%, whereas the proportion of people >60 versus adolescents and young adults aged 10 to 24 will be 2.1 versus 2.0 billion.^3,4^

This is going to create a need for house environment interventions, aiming to serve the physical, psychological, and emotional well-being of the elderly, while helping them maintain or regain their independence and autonomy, all of which promote longevity.^3,4^ Thus, developed countries are expanding on both the typology of their homes^5–7^ for aged individuals, and services they offer for people with long-lasting debilitating chronic diseases such as Alzheimer's disease.^8–10^

However, similar problems arise while facing the abrupt onset of a debilitating and long-lasting health condition, even among younger populations. In this setting, debilitated individuals and their families have limited time to find a new home or make radical structural modifications in their residence. Consequently, there is a question of how the affected members can carry on living with their families, while meeting their own emerging needs for specialized facilities, required throughout their daily routine; the time factor is also to be considered, given that these living arrangements may be needed for an interval ranging from months to years.^11,12^

Of note, the onset of debilitating clinical conditions (due to cancer or a rapidly evolving myopathy, neuropathy, respiratory disease, or a severe traumatic injury) is generally not expected among young and middle-aged individuals; therefore, housing and household necessities cannot be arranged in advance.

In this article, we attempt to perform an explanatory literature review of published articles applicable to the aforementioned problematics, aiming to estimate the current level of relevant awareness and communication, among patients, their families, and health professionals.

## Methods

We performed a scoping literature review, searching PubMed database by the following key words: “interior design,” “debilitating conditions,” “home care,” “end of life care,” “care of advanced cancer patients,” “care of patients with mental disorders,” “home care of covid-19 affected patients,” and “care of patients with degenerative illnesses.” We selected articles published between 2000 and 2020, focusing on clinical trials and reviews. We did not exclude articles published in non-peer-reviewed medical journals. We ended up including 24 articles.

## Results

### Introduction

Professional assessment of housing conditions of debilitated patients has already been established. For example, in the United Kingdom, debilitated patients are systematically assessed by occupational therapists, to suggest the housing modifications that may be needed, leading to favorable outcomes for the affected patients.^13^ In this same context, it has been suggested that alternative interior design techniques may provide timely and effective solutions without permanently impacting the mural house domains. But at which pace can these designing solutions be employed? Internal architecture designers may promptly provide solutions once a particular need is identified, as it happened in the cases of COVID pandemic and mental disorders.^14–19^

### COVID-19 pandemic

In 2020, the COVID pandemic brought about unprecedented changes to humanity. The long-lasting no-gather and stay-at-home guidance, as well as self and environmental measures,^14,15^ led to collectively spending far more time in-house than we had ever anticipated, with notable impact on home-working, home-living spaces, and psychological well-being. Self-isolation for individual protection from a confirmed case in the same household was and remains an ongoing challenge for 2021. For these reasons home internal design measures had been suggested by specialized professionals. Environmental hygiene was thereafter promoted with the use of antibacterial materials with hygienic properties (copper, brass, and bronze materials, quartz, and wood from bamboo, oak, and cork). The needs of psychological well-being, working at home, and quarantining can be answered by separating areas and creating sanitary spaces offering a sense of security, calmness and relaxation, covering all the aforementioned needs.^16–18^

### Mental disorders

Mental disorders represent a major medical and social issue, frequently correlated with severe distress, functional disabilities, and heavy economical burdens. The impact of interior design interventions on schizophrenia, anxiety, stress-related disorders, bipolar disorder, depression, and dementia has been explored in various reviews and clinical trials.^19–22^ Minimalistic interior design, with removal of attention distracting objects, hiding or stressing key features, regulating sunlight exposure, space for practicing yoga, and roofs allowing the observation of the outside landscape have been employed to facilitate the healing of mentally ill patients.^19–22^ Nonetheless, there is a lack of systematic research, thus the available data are scarce, and fail to cover the whole spectrum of how various environmental factors may affect mental disorders.^20^

### Cancer

Other interesting research recently evaluated some interior design applications to improve the quality of life of cancer patients living in two floor houses. Although duplex houses constitute a rapidly developing market and have become quite popular as they combine a better compartmentalization of living space with luxurious appearance, problems may emerge if the residents suffer from a debilitating condition. Patients with advanced cancer, muscle weakness, or cachexia might find the use of stairs connecting the bedroom (first floor) to the living room (ground floor) challenging, thus isolating themselves in the bedroom, without being a part of daily family life, which can cause emotional distress or even depression. An interior design intervention at the living floor may improve patient's daily routine, mood, performance, and quality of life without expensive or permanent structural interventions to the house.^23^

## Discussion

There is limited evidence on the impact of interior design in the treatment of other debilitating conditions. It might be assumed that the science of interior architecture may positively impact other important maladies of neurological, respiratory, and traumatic origin offering a relief not only for the patients but also for the entire families living in the same environment. Nonetheless, the medical world is still unaware of these possible solutions, leading to communication gaps between medical and interior architecture experts, concerning patients' needs.

As patients and relatives may not be aware of interior design solutions for the new daily life requirements and care resulting from their condition, interior design experts may equally be unaware of who is in need of their expertise solutions. Thus, an intermediary is needed to introduce patients to interior design solutions. Interdisciplinary collaboration between interior designers and occupational therapists is expected to further benefit patients/those needing house interventions.^24^ Occupational therapy feedback can be used to guide interior designers to better understand the anatomical needs imposed by the new debilitating condition.

Since the goal of supportive care is to improve patients' well-being and prevent or promptly treat the disease symptoms, side effects caused by treatments, and psychological, social, and spiritual problems related to a disease or its treatment, the supportive care working group is probably the most appropriate “specialty” to address this issue and bridge the gap between medical and interior design sciences. Residential architectural barriers and impediments need to be rapidly dealt with either at the hospital during a supportive care visit (using a small dedicated self-administered questionnaire), or at home by health personnel if domiciliary supportive care is delivered (taking into account that home-care support and facilities are not omnipresent).

## Conclusion

To conclude, the interaction between health and interior space architecture is scarce. Bridging interior architecture and medical science may offer novel possibilities in meeting patients' needs. In this context, a greater degree of quality collaboration and communication between health and interior design professions might facilitate the development of in-home solutions for meeting the needs and challenges of patients with debilitating health conditions.
